# Intestinal Antisepsis1Read before the Illinois State Veterinary Medical Association, December 2, 1902.

**Published:** 1903-05

**Authors:** J. H. Crawford

**Affiliations:** Harvard, Ill.


### INTESTINAL ANTISEPSIS.[1]

By Dr. J. H. Crawford,
HARVARD, ILL.

A MORE appropriate name might have been chosen, but, as Shakespeare says, "What is in a name?" Therefore, in introducing the subject, we will consider it in three different phases : (1) Why we should use them. (2) What we expect to accomplish by their use. (3) Which of the numerous antiseptic agents can we safely use with a fair prospect of obtaining results?

Chemical investigation has shown that many diseases depend upon the products of putrefaction and fermentation, rather than upon the direct action of microbes upon the tissues. These products are called ptomaines, which resemble vegetable alkaloids. These alkaloids, however, are not all poisonous; the poisonous ones are termed toxins. And it is those with which we have to deal. As toxins owe their development to the micro-organisms,

[1] Read before the Illinois State Veterinary Medical Association, December 2, 1902.

it follows that toxins formed depend on the material acted upon, the conditions under which the putrefaction goes on, and probably the health of the animal in whose body these processes are taking place. Some bacteria require oxygen, others do not; consequently the toxins manufactured by those two classes of organisms differ very materially. Metabolism is taking place everywhere within the body, with the result that the complex molecules of brain and muscle in their catalysis pass through intermediate stages, and are finally resolved into carbonic acid, water, and ammonia. We do not know what part oxygen plays in the processes of putrefaction; but the researches of Pasteur have shown that bacteria play a very important part in the disintegrating processes of organic matter, and in no part of the body is this more true than in the intestines.

Self-poisoning from the absorption of toxic substances secreted in the intestines is only prevented by the activity of the excretory organs of the body, chiefly the kidneys and liver, the liver acting the part of a sentinel to the material brought by the portal vein from the alimentary canal.

Therefore, when we consider the amount of toxic materials in the alimentary tract of a diseased animal, when suffering from any of the infectious or contagious diseases or any of the various digestive troubles, particularly where there is considerable fermentation going on, also in diarrhœas, parturient paresis, rheumatism, laminitis, and other diseases too numerous to mention, it can be readily seen that the use of intestinal antiseptics in such cases are not only necessary, but imperative.

We will pass on to the second consideration—that is, what we expect to accomplish by the exhibition of antiseptics in the alimentary tract.

Undoubtedly the first indication is to get rid of the material which gives rise to the putrefactive and fermentative processes. This is most readily accomplished by inducing catharsis. Unfortunately, this is not always a safe procedure in the horse. The next best thing is to come as near that point as possible, by dieting and by the use of the proper laxative remedies, such as the condition indicates. On the other hand, free catharsis may be induced by eserine, arecoline, barium, aloes, aloin, oils, etc. Having cleared out the alimentary tract, our antiseptics are now in order. Having selected our remedies according to the conditions present, by their use we expect to prevent, as far as possible, the formation of various toxins and gases; in other words, to get the alimentary tract in as aseptic a condition as we can, and keep it so. Of course, we understand that it is absolutely impossible to get the digestive tract in a purely aseptic condition, but there is no doubt that a great deal can be accomplished by the administration of antiseptic remedies, and, in so doing, we will undoubtedly modify to a large extent the pathological processes induced by the absorption of toxins into the system.

We now come to the point where we have to select remedies, and in our selection we must be careful to administer only drugs that are reliable, and in doses that are not in themselves poisonous; for in killing the microbes we must not destroy the patient. At the same time, we know that the best way to disinfect the stomach and intestines is to restore them to their normal condition. Among the large numbers of antiseptic agents at our disposal are the various members of the coal-tar group, such as carbolic acid, creolin, salol, creosote, naphthalin, and beta-naphthol. There are also bismuth salicylate, salicylic acid, iodoform, boric acid, quinine, charcoal, and there are also the various mercurial salts, which are all antiseptic and also more or less poisonous.

However, we may combine several of these agents, and out of them get a fairly reliable and safe antiseptic remedy. Of late I have used a remedy composed after the following formula:

| | | |
|---|---|---|
| Carbolic acid | | ℥iij. |
| Boric acid | | ℥iv. |
| Oil of gaultheria | | ℥j. |
| F. E. capsicum | | ℥iss. |
| Glycerin | | ℥viij. |
| Alcohol | | Oiij. |
| Aqua | q. s. | Ov. |

This is given in doses of from one to two ounces and can be repeated as necessity requires. The above formula is open to criticism, no doubt. It may be called conglomerate, shot-gun, or anything else. It may also be said that as good or better results can be obtained by one single drug. That may be so in any one disease by selecting the drug best suited to that disease; but for a good general intestinal antiseptic the above has proven successful in my practice, and a careful summing up of the various actions of the drugs used will substantiate that proposition. Expense is also a very important point on account of the large doses used. On that point it fills the bill admirably. It can be used in colics, the pneumonias, and, in fact, in all febrile or digestive troubles, as a general intestinal antiseptic.

In conclusion, I would say that by the presentation of this paper I hope to bring out a good, sharp discussion that we may all profit by, and that it may stimulate us to give the subject of intestinal antiseptics more thought, and by so doing evolve new ideas that may redound to the benefit of veterinary science and veterinary practitioners in general.

